# Conductive Hydrogel Electrodes for Delivery of Long-Term High Frequency Pulses

**DOI:** 10.3389/fnins.2017.00748

**Published:** 2018-01-11

**Authors:** Naomi A. Staples, Josef A. Goding, Aaron D. Gilmour, Kirill Y. Aristovich, Phillip Byrnes-Preston, David S. Holder, John W. Morley, Nigel H. Lovell, Daniel J. Chew, Rylie A. Green

**Affiliations:** ^1^Graduate School of Biomedical Engineering, University of New South Wales, Sydney, NSW, Australia; ^2^Department of Bioengineering, Imperial College London, London, United Kingdom; ^3^Medical Physics and Biomedical Engineering, University College London, London, United Kingdom; ^4^School of Medical Science, University of New South Wales, Sydney, NSW, Australia; ^5^School of Medicine, Western Sydney University, Sydney, NSW, Australia; ^6^Galvani Bioelectronics, Stevenage, United Kingdom

**Keywords:** conductive hydrogel, high frequency stimulation, nerve block, neural interfaces, peripheral nerve cuff array

## Abstract

Nerve block waveforms require the passage of large amounts of electrical energy at the neural interface for extended periods of time. It is desirable that such waveforms be applied chronically, consistent with the treatment of protracted immune conditions, however current metal electrode technologies are limited in their capacity to safely deliver ongoing stable blocking waveforms. Conductive hydrogel (CH) electrode coatings have been shown to improve the performance of conventional bionic devices, which use considerably lower amounts of energy than conventional metal electrodes to replace or augment sensory neuron function. In this study the application of CH materials was explored, using both a commercially available platinum iridium (PtIr) cuff electrode array and a novel low-cost stainless steel (SS) electrode array. The CH was able to significantly increase the electrochemical performance of both array types. The SS electrode coated with the CH was shown to be stable under continuous delivery of 2 mA square pulse waveforms at 40,000 Hz for 42 days. CH coatings have been shown as a beneficial electrode material compatible with long-term delivery of high current, high energy waveforms.

## Introduction

Recent studies have indicated that electrical therapies, in particular nerve block, may be an effective treatment for chronic conditions such as inflammatory bowel disease, arthritis, asthma, and diabetes (Famm et al., 2013; Birmingham et al., 2014; Langdale et al., 2017). These are disease states where current pharmaceutical approaches have been effective with a large number of patients, but in patients with persistent, non-responsive or resistant cases, treatment options are limited. Recent efforts have therefore focused on engineering a device capable of delivering a flexible range of stimulation, recording and nerve block paradigms for application to the peripheral nervous system (PNS). Such a device is expected to be necessary for tuning system requirements to peripheral nerve fibers within a fascicle and ensuring the health of off-target tissues both adjacent to the device and within, but distal to the neural branch being targeted (for example, organs that are innervated by the same nerve but not the therapeutic target). While hardware specification, neuromodulation techniques, and biological mapping of visceral nerves are key areas that must be developed, an interfacing device, capable of fitting the many variable and non-uniform fibers of the PNS must also be designed. As specified by Birmingham et al. (2014), new materials and architectures are required to address the largely unmyelinated nerve fibers, irregular neuroanatomy, and movement in the viscera.

Critical to neuromodulator devices is the electrode array, used to interface with and control spatially selective activity within a nerve. To achieve control of a physiological process through nerve modulation within the PNS, it is essential to steer electrical current toward the correct nerve fiber(s) (Lovell et al., 2010). Nerves within the PNS consist of both afferent and efferent fibers that extend to innervate the organs of the body. Afferent fibers carry signals toward the brain, while efferent fibers take signals from the brain to the various peripheral organs and muscles. In an application where organ control is required, such as asthma or diabetes, it is necessary to target the efferent neurons. As most neuro-immune pathways and organ systems are closed-loop, they are reliant on a balance of both afferent inputs and efferent outputs (Pavlov and Tracey, 2017). Inadvertent blocking of the afferent nerves may result in an undesirable/inappropriate response from the brain, ultimately exacerbating the condition being treated. As such, it is critical that an electrode array used for nerve block, can deliver modulating signals to specific areas within the nerve across chronic time frames. One of the most significant challenges to development of such an array, is the need to create a stable electrode-neural interface where the capacity to deliver appropriate and targeted signals does not change over time (Guo, 2016).

A number of electrode array formats have been investigated for application to the PNS, and these are broadly categorized as penetrating and non-penetrating arrays. Penetrating arrays have been shown to be beneficial for spatially selective activation of nerve fibers, being placed within the nerve bundle, beneath the perineurium, and hence closer to the target tissues (Lago et al., 2007; Boretius et al., 2010; Wark et al., 2013). Intrafasicular arrays are specifically designed to sit within the nerve fascicle. However, damage to the perineurium during implantation has been associated with a number of negative consequences, including increased endoneurial pressure, nerve fiber compression, and loss of nerve fibers (Grill et al., 2009). As the neural wound attempts to recover, the chronic presence of a stiff device comprised of foreign material within the nerve, results in growth of fibrous scar tissue that can isolate the electrode arrays and negate the benefit of proximity (Bowman and Erickson, 1985; Zheng et al., 2008). Conversely non-penetrating or cuff arrays, are designed to wrap around the nerve, minimizing damage to the native tissue, but inherently having less spatial selectivity for targeting specific nerve fibers (Tyler and Durand, 2002; Grill et al., 2009). Regardless of the format, these devices are commonly fabricated from conventional electrode array materials, with metallic contacts embedded in polymeric insulators. Some newer approaches based on carbon fibers have been designed to be more flexible, lower profile and biocompatible (Gillis et al., 2017), but the long term *in vivo* performance of these fibers remains unknown.

There are two significant challenges associated with interfacing an electrode array with the visceral nerves of the PNS, one is mechanical and the other is electrical. Regardless of format and placement, there is an inherent mechanical mismatch in the interaction of a relatively stiff electrode array with soft nervous tissues (Green et al., 2008; Grill et al., 2009; Guo, 2016). This mismatch is exacerbated by the substantial displacements of PNS nerve fibers associated with human movement, and can result not only in dislocation of the device but associated damage to the surrounding neural tissue (Grill et al., 2009; Birmingham et al., 2014). Damage and the presence of foreign materials generate inflammatory reactions that lead to fibrous encapsulation of a device. Both the movement of the array and scar tissue encapsulation have resulting impacts on the electrical properties of the neural interface, making spatially selective neuromodulation difficult and unstable over time. The increase in distance between the device and target nerves ultimately leads to the need for application of higher currents to achieve a therapeutic response, at which point, conventional metal electrodes can suffer from electrically-mediated corrosion. This occurs due to high voltages being generated at the electrode-neural interface that cause adverse chemical reactions (including the generation of gases, H^+^ ions, and metal redox reactions that result in dissolution). While nerve block waveforms are expected to be in the high frequency range of neuromodulation and the short pulse durations minimizes the time for which a given current is applied and thus a chemical reaction can propagate, the usual techniques for preventing electrical imbalances in the system are not effective. For example, sensory neuroprosthetics often use in line capacitors to block DC currents from being transduced from device to tissue (Cogan et al., 2016b). An alternative approach is to short the electrodes in the array together between stimuli (Wong et al., 2009; Cogan et al., 2016b). Both of these techniques enable the device and tissue to maintain zero net charge and arrest any chemical reactions that may be present. However, at high frequency blocking capacitors are not effective (as they behave as a short rather than open circuit) and there is no interstimulus delay in which to equilibrate the system. As such chronic application of high frequency signals involving nerve block currents can lead to electrode damage and failure where charge balance is not perfectly preserved. This is of particular risk for metallic electrodes where the voltage produced at the interface is high and localized defects in the metal surface can initiate areas of charge imbalance and corrosion.

Conductive hydrogels (CHs) have been shown to be an effective material for mediating the mechanical properties of an electrode and simultaneously improving the electrical properties. CHs are a hybrid material produced from a conductive polymer (CP) and a hydrogel, with a mechanical modulus more than three orders of magnitude below that of platinum (Pt), a conventional bioelectrode material (Green et al., 2012b; Goding et al., 2017). Due to the presence of the hydrogel component, CHs swell in aqueous environments, enabling ingress of ions and forming a three dimensional surface through which charge is transduced. The substantially higher charge transfer area introduced by the CH enables coated electrodes to inject greater amounts of charge at lower voltage than their uncoated metallic counterparts and hence enable devices with low power consumption (Kim et al., 2004; Sekine et al., 2010; Pan et al., 2012; Hassarati et al., 2014, 2016). In prior studies by Hassarati et al. (2014) it was shown that CH coatings on cochlear implants imparted a reduction in potential transient of over 50% and maintained more stable electrical properties over a simulated 2 years of activity (two billion stimuli delivered continuously over 3 months in artificial perilymph). However, the capacity of these materials to deliver continuous high frequency pulses with relatively large amplitudes (mA as compared to prior studies with μA) is not known. Due to the efficiency in charge transfer of these materials, and their polymeric nature which imparts high electrochemical stability, it was proposed that CH coatings can provide a low voltage interface for delivery of a neural block. It was hypothesized that CH coatings could be used to provide stable, long-term performance of PNS nerve cuffs under high frequency stimulation.

While CH coatings have been routinely applied to Pt or platinum/iridium (PtIr) electrodes used in sensory stimulating neuroprosthetics, it was recognized that by changing the material that interfaces with the neural tissue, it may not be necessary to use a conventional electrode material as the substrate. Stainless steel (SS) has a history in implantable electrodes for recording and also macroelectrodes in cardiac pacing (Bowman and Erickson, 1985; Peixoto et al., 2009; Cogan et al., 2016a), however it is not commonly used in implantable neuroprosthetics. Recent studies by Aristovich et al. (2016) have demonstrated that these arrays are capable of delivering high frequency signals (>1.7 kHz) required for imaging neural activity by electrical impedance tomography (EIT). As an alternate and low-cost platform, SS electrode arrays were compared in this study to commercially available PtIr arrays for delivery of high frequency neural blocking waveforms, both with and without CH coatings. Both arrays types were non-penetrating cuffs, designed to wrap the nerve bundle without penetrating the fascicle. The commercial array was a two electrode pre-curled design, intended to facilitate easy placement around a nerve. The SS array was fabricated in a planar format with increased electrode density and resolution (30 electrode sites), designed for controlled current steering. These arrays were characterized before and after coating for charge transfer properties including maximum cathodic charge injection limit. An *in vitro* high frequency study was undertaken to establish the long term performance and robustness of each of the electrode types.

## Methods

### Conductive hydrogel fabrication

All conductive hydrogel coatings were fabricated from the same material components, however modification of protocol was required to tailor application to different array formats. All materials and reagents were obtained from SigmaAldrich unless otherwise stated.

The coating procedure was in line with prior literature on macroelectrodes and probe formats (Green et al., 2012b; Hassarati et al., 2014; Goding et al., 2017). This requires a three step protocol consisting of a pre-layer to improve coating adhesion to the underlying electrodes, formation of the non-conductive hydrogel layer and the polymerization of the CP component within the hydrogel to impart conductivity at the electrode sites.

The PEDOT/pTS prelayer was electrodeposited from a solution of 100 mM 3,4-ethylenedioxythiophene (EDOT) and 50 mM sodium p-toluenesulfonate dissolved in 1:1 deionized (DI) water (Baxter Healthcare Pty Ltd.)-acetonitrile solution. The hydrogel macromer solution consisted of 20 wt% poly(vinyl alcohol)-methacrylate-taurine (PVA-taurine) and 0.1 wt% Irgacure® 2959 dissolved in deionized (DI) water. PVA-taurine was synthesized in-house as described previously (Goding et al., 2017). The final step to produce a CH was electrodeposition from a CP solution of 30 mM EDOT and 0.3 mM NaCl dissolved in DI water.

### Coating of pre-curled commercial arrays

Commercial cuffs were purchased from Cortec Gmbh. Each cuff consisted of 2 electrode sites, where each electrode was formed by 2 connected pads being 0.7 × 1.15 mm each. The internal diameter of the pre-curled cuff was 1 mm. Cuff electrodes were immersed in PEDOT/pTS prelayer solution in a 2-electrode cell. A thin layer of PEDOT/pTS was galvanostatically deposited onto the PtIr electrodes using 1 mA/cm^2^ for 30 s and then rinsed with deionized (DI) water.

The hydrogel coating was applied by injecting 30 μL of hydrogel precursor into an opened cuff prior to closing the cuff around a glass capillary with a 0.7 mm outer diameter. Hydrogel was formed via photopolymerization using 30 mW/cm^2^ UV light for 180 s. The coated cuffs were soaked in DI water for 2 min prior to the removal of the glass capillary.

Finally, PEDOT was electrochemically deposited through the PVA-taurine coating immediately after photopolymerization. The coated cuff electrodes were immersed in CH deposition solution in a 2 electrode cell. PEDOT was galvanostatically deposited using 1 mA/cm^2^ for 10 min and then rinsed with DI water.

### Coating of planar SS arrays

Planar electrode arrays were fabricated from 313 L stainless steel with polydimethylsiloxane insulation. The electrode sites were 0.35 × 3 mm. These arrays were cleaned prior to coating, to remove oxides and residual debris from fabrication. The arrays were immersed in 1 M HCl for 2 min, and then sonicated in DI water for 5 min. This process was repeated prior to electrodeposition of the pre-layer. A thin layer of PEDOT/pTS was galvanostatically deposited onto the stainless steel (SS) electrodes using 1.5 mA/cm^2^ for 30 s and then rinsed with DI water.

The hydrogel coating was applied by pipetting 30 μL of hydrogel precursor onto the flat cuff. A coverslip was then used to push the solution into the recesses formed by the silicone insulation bordering the electrode sites. Excess macromer solution was removed from the surface of the array. The hydrogel was crosslinked via photopolymerization using 30 mW/cm^2^ UV light for 180 s.

Finally, PEDOT was electrochemically deposited through the PVA-taurine immediately after photopolymerization. The coated cuff electrodes were immersed in an aqueous 0.1 M EDOT solution in a 2 electrode cell. Ten electrode sites were shorted together to enable electrodeposition of multiple sites in parallel. PEDOT was galvanostatically deposited using 1 mA/cm^2^ for 10 min and then rinsed with DI water.

### Electrochemical characterization

EIS was conducted using an eDAQ Electrochemical Impedance Analyzer (Z100) in conjunction with an eDAQ Potentiostat (EA163) controlled with the use of Z100 Navigator Software (WonATech Co. Ltd.). Recordings were made using an isolated, leakless Ag/AgCl reference electrode and a platinum wire counter electrode. Measurements were made in 0.9 wt% saline (Baxter Healthcare Pty Ltd.). CH coatings were subjected to a 70 mV sinusoidal voltage amplitude across a frequency range of 10,000 to 1 Hz with a 0 V DC offset voltage.

CV was conducted using an eDAQ e-corder (ED410) in conjunction with an eDAQ Potentiostat (EA163) controlled with the use of EChem software package. Recordings were made in a three electrode cell using an isolated Ag/AgCl reference electrode and a platinum wire counter electrode. Samples were subjected to a cyclical stimulation voltage from −800 to 600 mV at a scan rate of 150 mV/s in 0.9 wt% saline, taking the integral of the 10th cycle to calculate the charge storage capacity (CSC).

### Charge injection limit

Charge injection comparison was performed in a three electrode cell identical to CV and EIS. Charge injection limit was determined using protocols previously established by Cogan et al. (2005). The limit was defined as the voltage required to reach the reduction potential for water. An in-house biphasic stimulator was used to deliver constant current, charge balanced pulses. Phase length was varied from 0.01 to 0.8 ms, based on standards from the prior literature (Cogan et al., 2005; Green et al., 2014) and also the need to characterize for high frequency stimulation behavior, which is best modeled by short phase length waveforms. The current was increased until the residual interphase voltage (E_mc_) reached −600 mV vs. Ag/AgCl (see Green et al., 2014 for definition and schematics of E_mc_ relative to the applied biphasic waveform). The charge delivered across a single phase at this point was regarded as the charge injection limit.

### Long-term delivery of high frequency pulses

High frequency stimulation was performed by application of continuous square pulses (charge balanced and net zero DC bias) to electrode pairs in saline. The system used for high frequency stimulation was a custom-built unit comprising an arbitrary waveform charge balance current source (Howland CCS) capable of delivering sine or square waves via four isolated stimulators. The pulse frequency was set to 40 kHz with a peak-to-peak amplitude of 2 mA (1 mA in the positive phase and 1 mA in the negative phase). The total voltage across electrode pairs was monitored daily for the first 2 weeks and then at least twice weekly to ensure there was no drift or DC leakage. Total voltage was defined as the addition of maximum positive and negative voltage (peak to peak voltage). The net voltage (difference between absolute negative and positive voltage) was used as an indicator of imbalance or drift in the waveform. On a weekly basis, electrodes were removed from the high frequency stimulation and characterized using CV and EIS metrics (as detailed in the above protocols). Any changes in performance or appearance of electrodes was recorded and examined where necessary.

## Results

**Figure 1:**
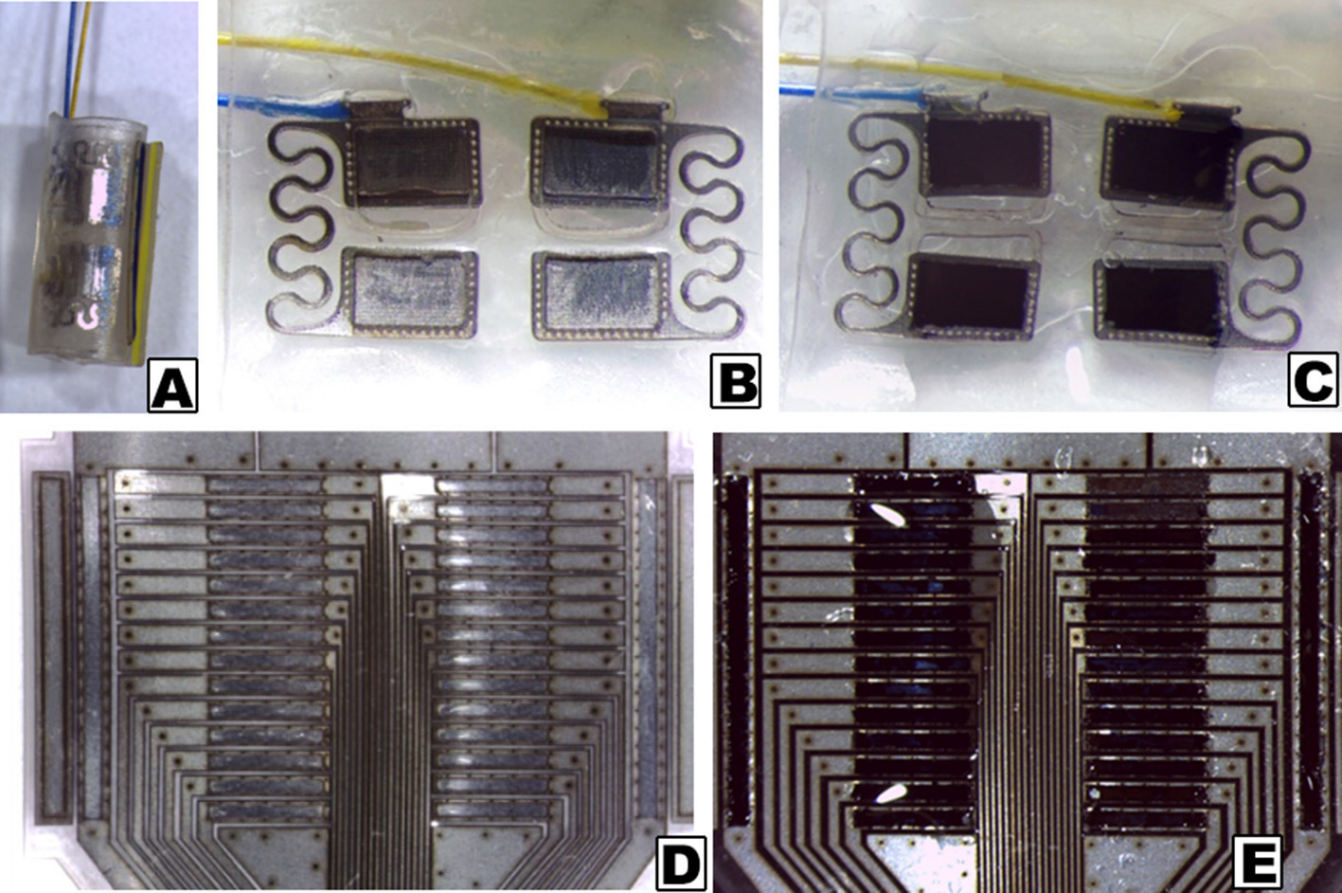
Stereoscopic images of electrode arrays, as received and with CH coatings. **(A)** Pre-curled commercial cuff array; **(B)** Opened cuff showing PtIr electrode sites without coating; **(C)** Opened cuff with CH coating on PtIr electrode sites; **(D)** Uncoated planar SS array; **(E)** CH coated planar SS array.

CH coatings were applied to both pre-curled and planar electrode arrays, as depicted in Figure 1. Due to the different formats, the pre-curled PtIr array was coated such that the entire inner (tissue contacting) surface was coated with a thin layer of PVA hydrogel (~100 μm). Subsequent electrodeposition of PEDOT resulted in conductive polymer growing in the discrete areas directly above the electrode sites. Parameters were controlled such that PEDOT was not grown beyond the electrode site boundaries, hence preventing bridging between electrodes. This contrasted to the planar array where the recessed electrode sites were filled with PVA hydrogel and subsequently PEDOT growth throughout this hydrogel layer (~50 μm). In this application the PEDOT is prevented from growing between electrodes as the hydrogel boundaries contain the conductive polymer growth.

### Electrochemical characterization

**Figure 2:**
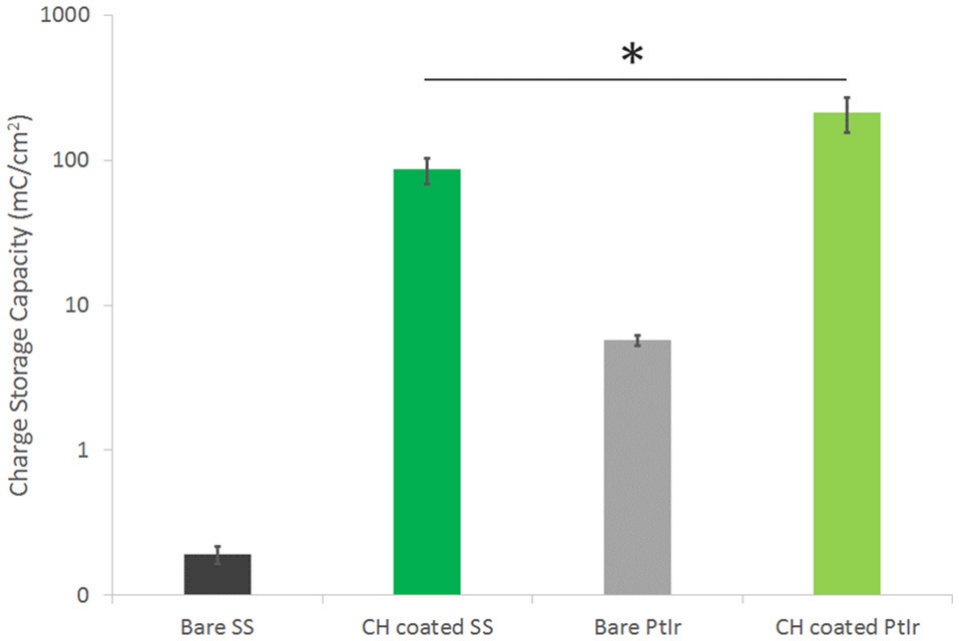
CSC of SS and PtIr electrode arrays before and after CH coating. Error bars are 1 *SD*, ^*^p < 0.05, (*n* = 8). Note log scale on y-axis required to enable SS data to be visualized.

Electrochemical analysis of each of these arrays was conducted prior to and after coating. The CV curves were integrated to yield CSC, as shown in Figure 2. The SS was found to have a CSC more than one order of magnitude lower than PtIr (0.48 mC/cm^2^ compared to 5.70 mC/cm^2^). The CH coating on the PtIr similarly had a significantly higher CSC (student *t*-test, *p* < 0.05) than that on the SS, although the difference was substantially lower (not an order of magnitude). The CH coated SS had an average CSC of 85 mC/cm^2^ and the CH coated PtIr was recorded as having an average CSC of 212 mC/cm^2^. Both CH coatings improved charge transfer by at least two orders of magnitude on their respective substrates.

**Figure 3:**
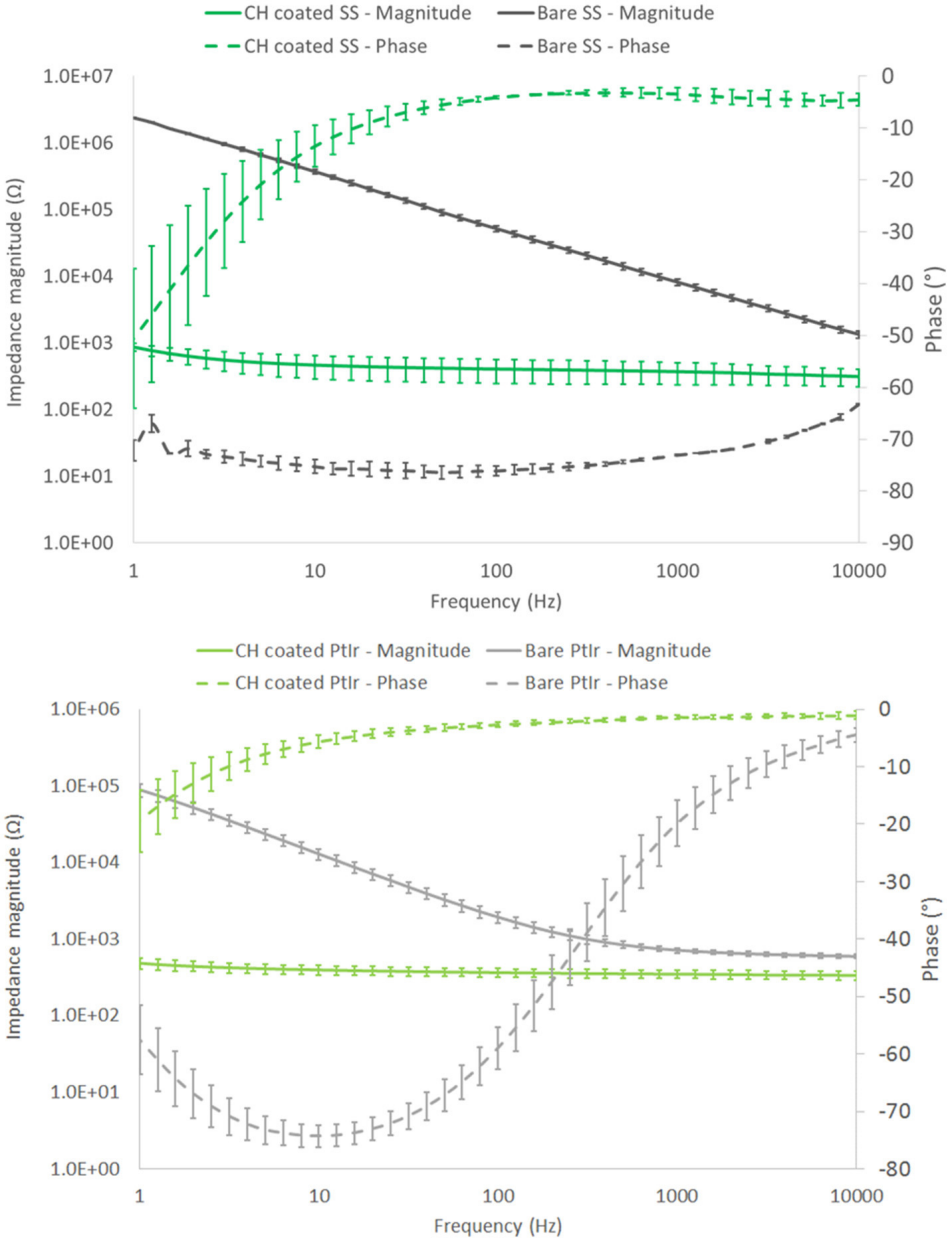
Frequency dependant response of SS **(top)** and PtIr **(bottom)** nerve cuff electrode arrays. Both bare and CH coated arrays are characterized. Error bars are 1 SD, (*n* = 8).

EIS verified that similar electrochemical performance was observed when using frequency dependant analyses. As depicted in Figure 3, SS had a significantly higher impedance at low frequency (around 2 MΩ @ 1 Hz) compared to the PtIr (average of 89 kΩ @ 1 Hz). However, these electrodes do differ in size, and when normalized for geometric charge transfer area have an average impedance of 24.9 and 1.5 kΩ.cm^2^ at 1 Hz for SS and PtIr respectively. As frequency is increased the impedance of the SS electrodes decreased to 85.6 Ω.cm^2^ at 1 kHz and 14.1 Ω.cm^2^ at 10 kHz. Comparatively the PtIr impedance decreases to 11.8 Ω.cm^2^ at 1 kHz and further reduced to 9.8 Ω.cm^2^ at 10 kHz. It is clearly seen that the SS has a different electrochemical behavior to the PtIr, with capacitive behavior dominating across the entire frequency spectrum. SS has been reported in prior studies as having variable EIS response that is dependent on both degree of passivation and alloy content (Wallinder et al., 1998).

When these arrays were coated with the CH the SS coated arrays experienced an average impedance magnitude of 865 Ω (9.1 Ω.cm^2^) at 1 Hz, reducing to 312 Ω (3.3 Ω.cm^2^) at 10 kHz. The CH coated PtIr recorded an average impedance magnitude of 497 Ω (8.0 Ω.cm^2^) at 1 Hz, reducing to 346 Ω (5.7 Ω.cm^2^) at 10 kHz. As such there was no significant difference in frequency dependant impedance performance of the CH on PtIr in comparison to CH on SS. Both arrays experienced significant reductions in impedance compared to their uncoated control arrays across all frequencies, including the high frequency range in which neuromodulation by blocking is expected to be performed (Famm et al., 2013).

### Charge injection limit

**Figure 4:**
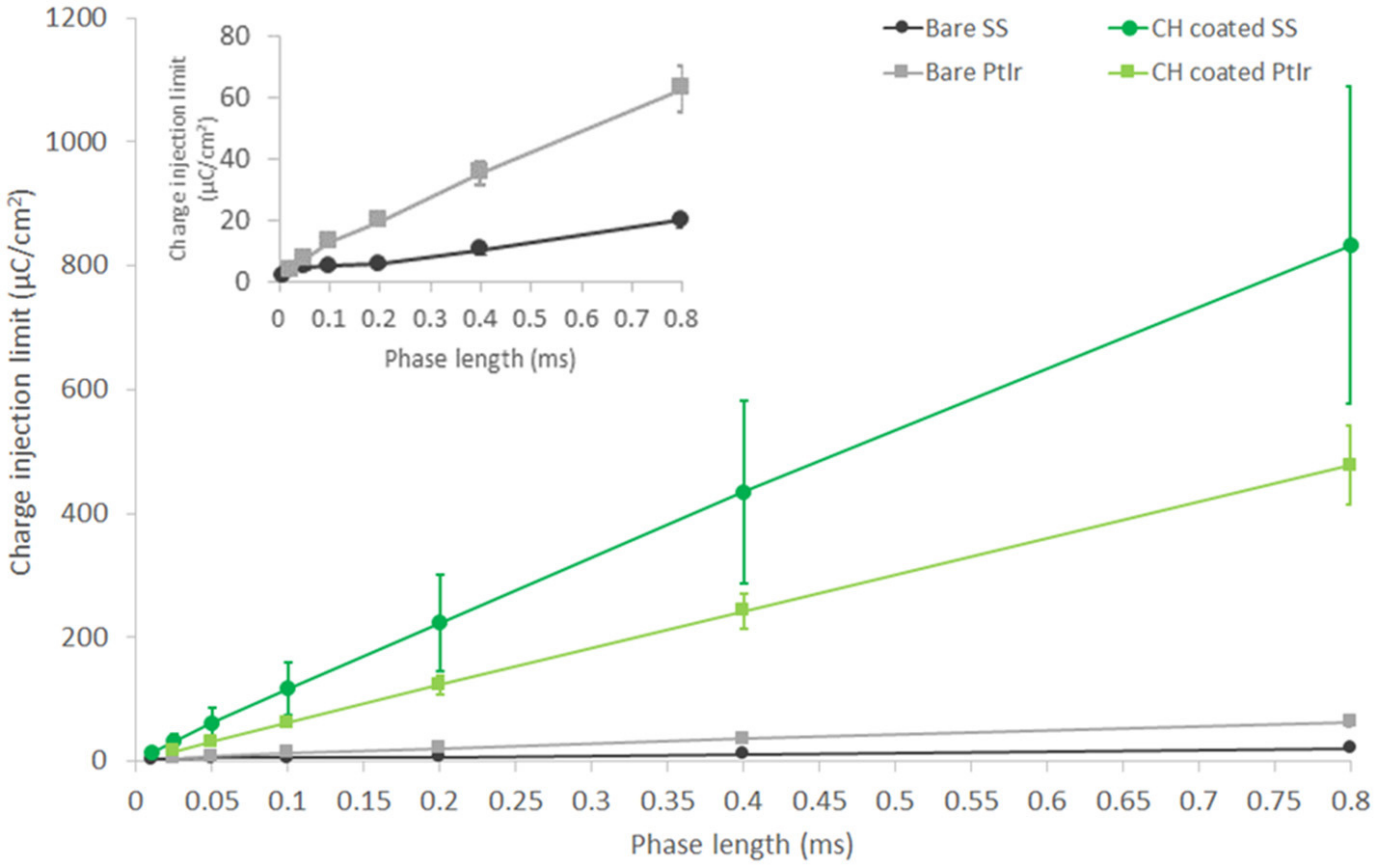
Charge injection limit for both PtIr and SS nerve cuff arrays, bare, and with CH coatings. Inset figure shows charge injection behavior of bare PtIr and SS at short phase lengths. Performed in saline. Error bars are 1SD (*n* = 8).

Charge injection limit studies were performed in saline to establish a maximum current that can be passed by each electrode material before electrochemical reactions associated with irreversible Faradaic reactions are enabled. It should be noted that this characterization technique is based on biphasic stimulation and involves the measurement of residual voltage at the electrode interface between cathodic and anodic pulses. As such it is not directly applicable to high frequency nerve block paradigms, however it does provide a metric for comparison related to application of neuromodulation devices, and will also provide guidance on the relative impacts of DC leakage current or drift in the applied nerve blocking waveform. The prior electrochemical analyses are not as well aligned with in-use electrode performance, being related to application of ramped voltage across long time courses (for CV) and application of a wide range of stimulation frequencies (for EIS). As shown in Figure 4, CH coatings were able to substantially improve the electrochemical charge injection limit of both electrode types. As with other metrics, it can be seen that the SS arrays are not able to inject levels of charge commensurate with the PtIr at longer phase lengths, until they are coated with the CH. While it is clear that the CH coated SS has the highest charge injection limit at higher phase lengths, the short phase length is most applicable to high frequency stimulations. A 40 kHz square wave has an effective phase length of 0.025 ms. Using this short phase length the CH coated PtIr has an injection limit of 15.8 ± 1.8 μC/cm^2^ and the CH coated SS has an injection limit of 13.5 ± 5.1 μC/cm^2^. The uncoated controls have injection limits of 4.1 ± 0.1 μC/cm^2^ for the PtIr and 2.4 ± 0.3 μC/cm^2^ for the uncoated SS.

### Long-term delivery of high frequency pulses

**Figure 5:**
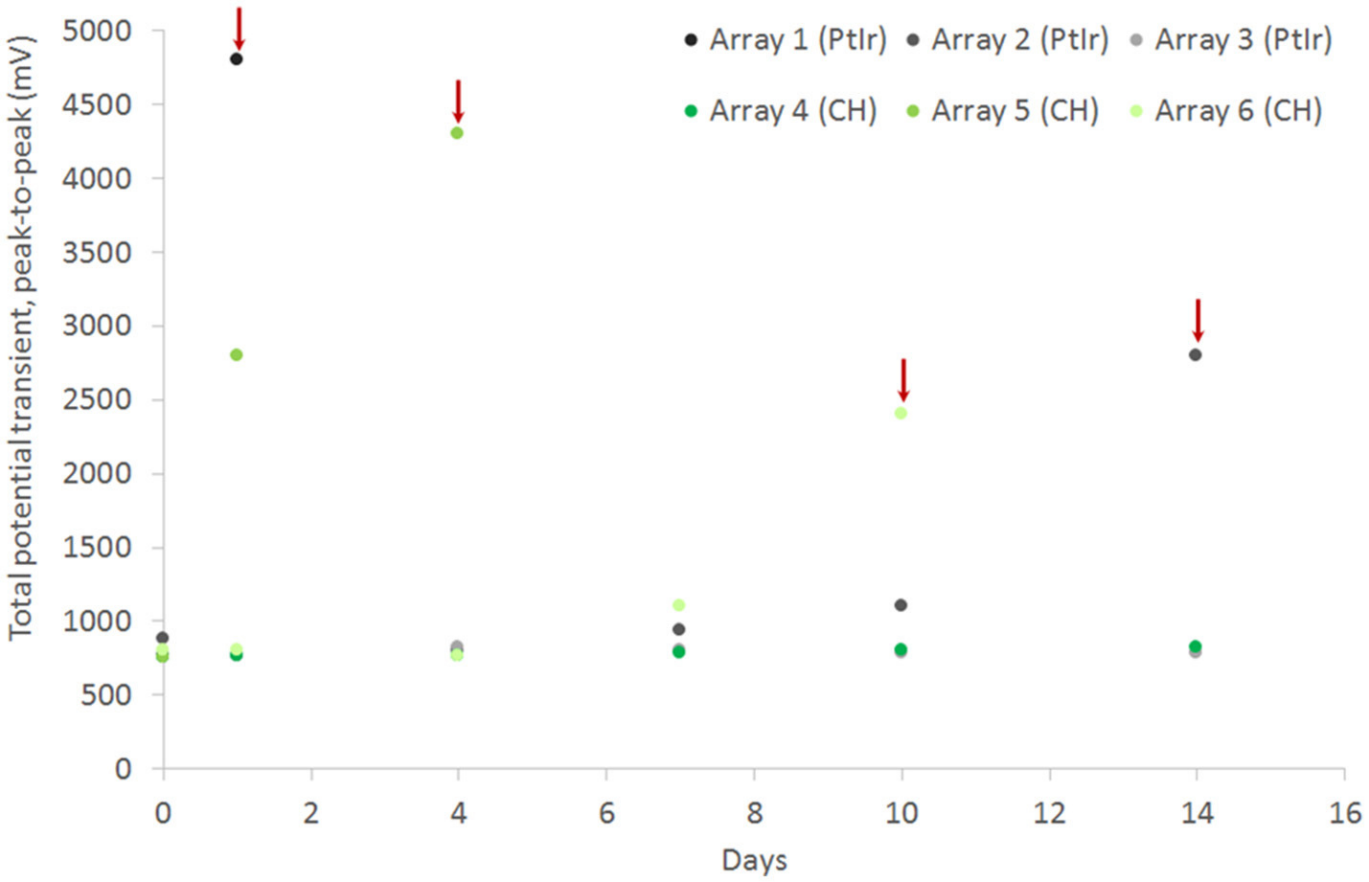
Total voltage drop across commercial PtIr electrode pairs under continuous high frequency stimulation, comparing performance for both bare electrodes and CH coated electrodes. Red arrow indicate an electrode pair that is considered to have failed due to sudden increase in potential transient.

**Figure 6:**
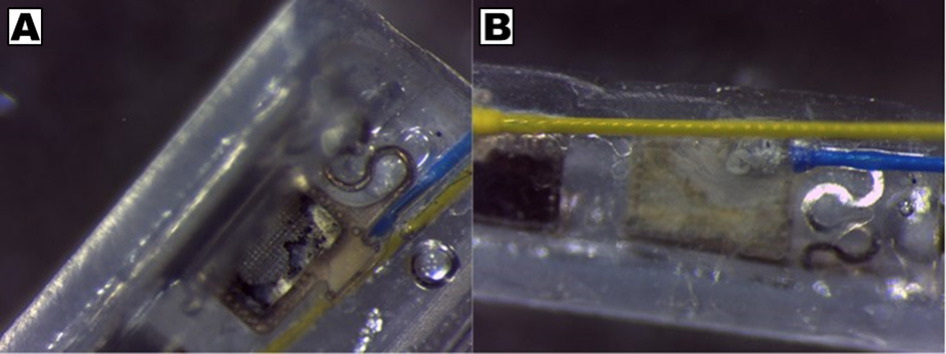
Corrosion of wire and pad connections on PtIr pre-curled cuff arrays, showing **(A)** delamination of CH coating and **(B)** presence of discolored precipitate at bonding point on back of electrode sites.

**Figure 7:**
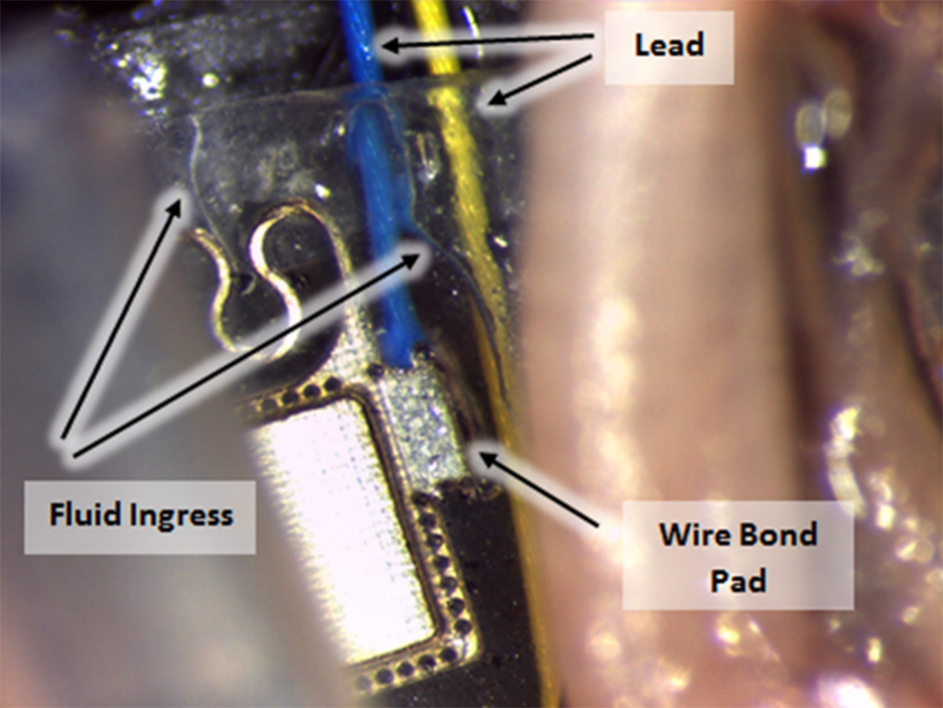
Fluid ingress to commercial cuff connection points, enabling corrosion with continuous electrical stimulation.

CH coating of electrodes was successful and able to improve charge transfer characteristics of arrays irrespective of underlying material type, however the stability and robustness of the system is critical for application in implantable bioelectronics. To understand the operational lifetime and limitations of coated electrodes an *in vitro* test was designed. This assay was based on prior studies that used continuous high frequency stimulation as an accelerated electrical test for coatings on cochlear implants and planar bionic eye electrode arrays (Green et al., 2012a; Hassarati et al., 2014). Due to the restricted channel numbers on the stimulator the commercial arrays and planar arrays were tested in separate studies. The commercial PtIr cuffs were found to have limitations when exposed to continuous high frequency electrical pulses. Initially the bare PtIr electrodes produced an average peak-to-peak voltage of 980 mV and the CH coated PtIr produced an average of 720 mV. However, with continuous stimulation both bare PtIr and CH coated PtIr were found to experience large increases in peak-to-peak voltage, as seen in Figure 5. Inspection of these electrodes revealed that failure was occurring at the connection where the wire was bonded to the electrode pad (see Figure 6). It should be noted that these commercial cuffs are designed for recording peripheral nerve activity, and as such this application is outside of their typical specification for use. Further investigation revealed that limited cohesion between both the PtIr electrode sites and the laminated silicone insulation led to fluid ingress around the bonding sites, as depicted in Figure 7. Due the presence of dissimilar metals and an ionic fluid environment, it is not surprising that some chemical corrosion was able to propagate. Where wire corrosion resulted in high voltage at the bonding point it was found that formation of gas beneath the CH coating resulted in delamination of this material. For both coated and uncoated arrays 2 of the 3 samples failed prior to 14 days and as such this study was not continued on this array format.

**Figure 8:**
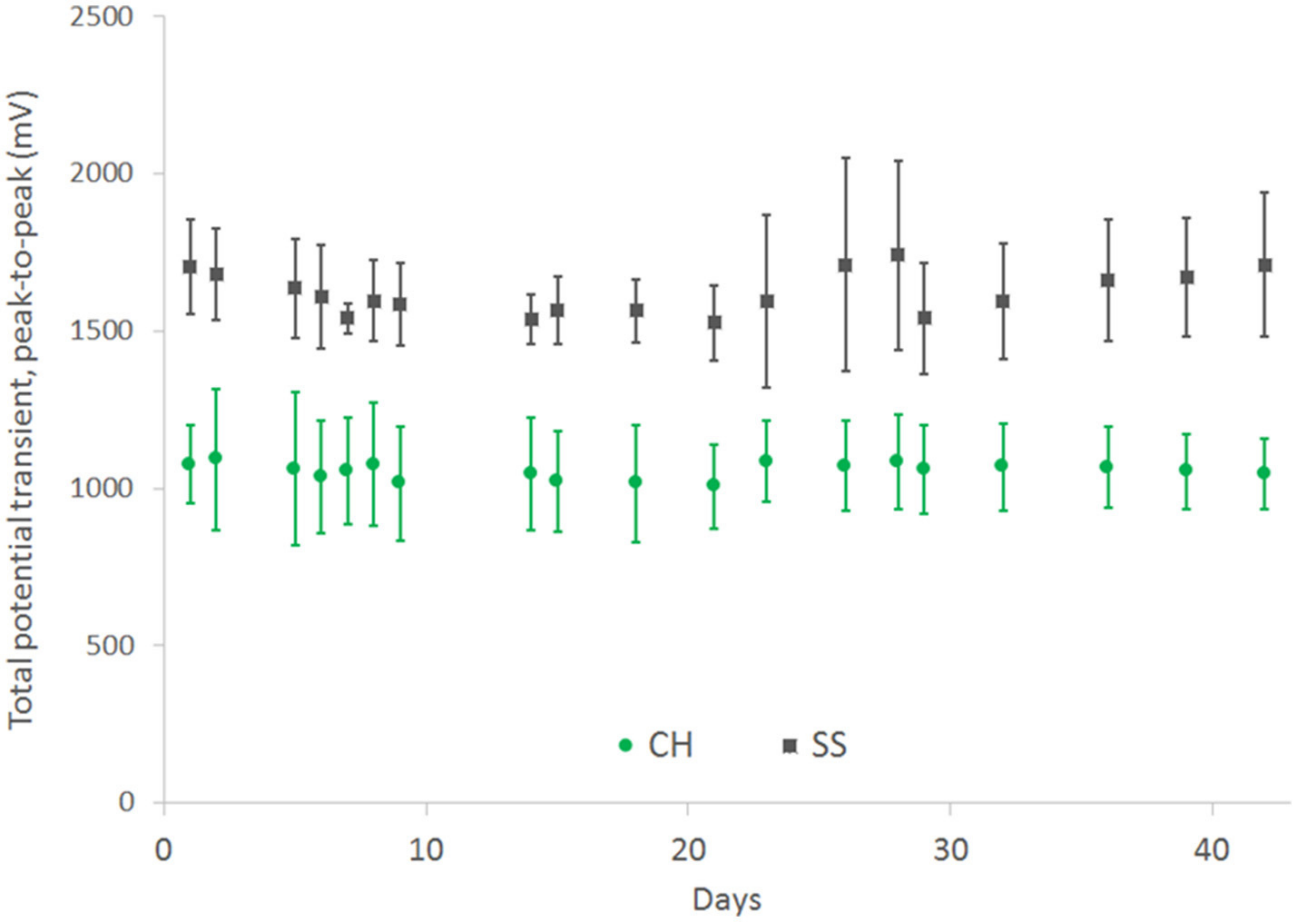
Total voltage drop across SS electrode pairs under continuous high frequency stimulation, comparing performance for both bare electrodes and CH coated electrodes. Error bars are 1 SD (*n* = 4).

In comparison the SS cuffs were found to have greater stability, although the connections for this array were significantly more distant from the electrode sites and were not immersed in the saline electrolyte. The total potential transient on the CH coated arrays was stably maintained over the 42 day study period at a value that was 33% lower than that of the uncoated controls (Figure 8).

**Figure 9:**
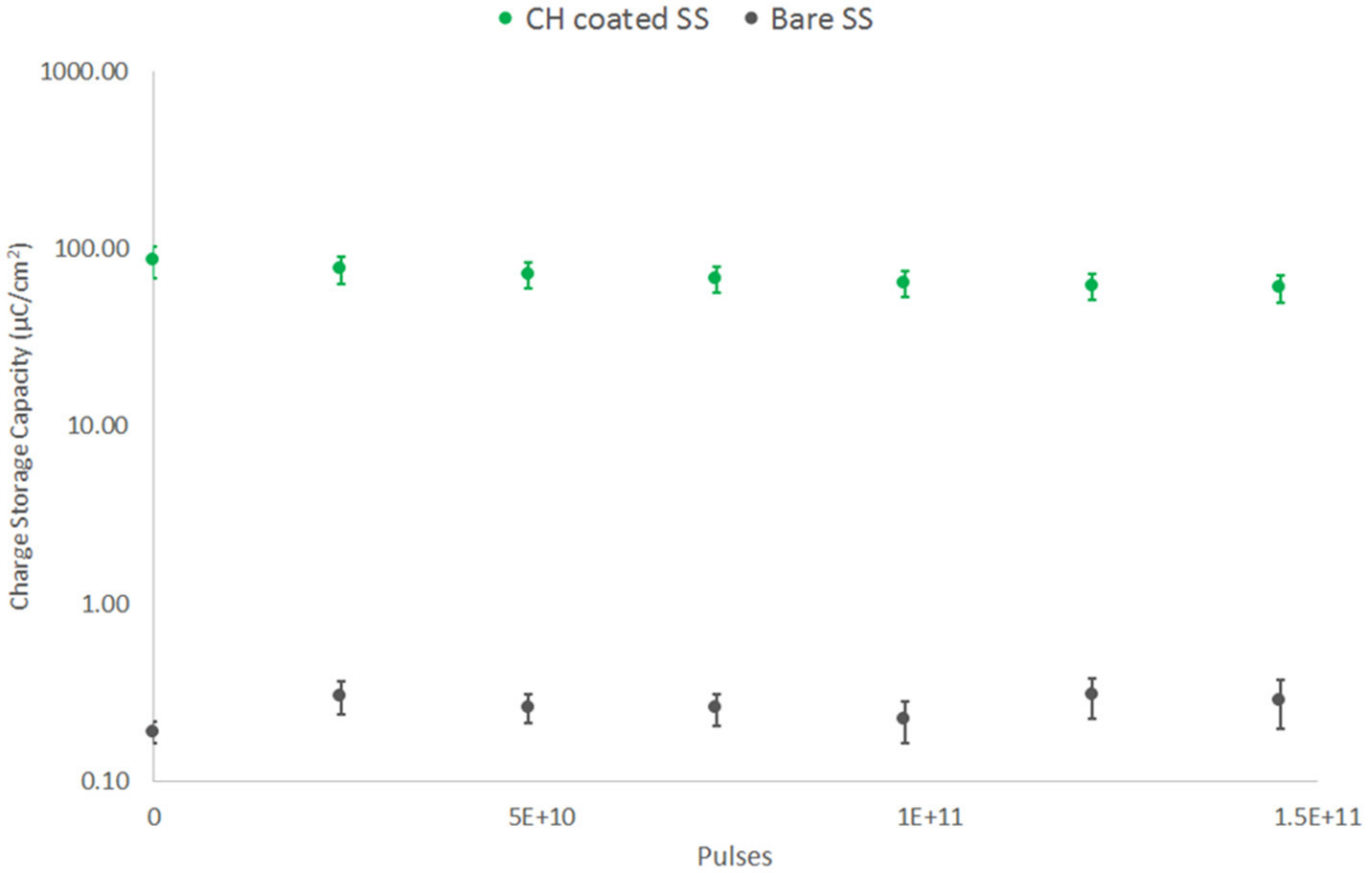
CSC of SS electrodes under high frequency stimulation (40 kHz, 2 mA peak-to-peak). Comparison of CH coated SS and bare SS electrodes over 42 days or 150 billion pulses (*n* = 8).

The electrochemical characteristics of these electrodes were monitored weekly. As shown in Figure 9, there was no significant change in CSC across the 42 day testing period for either electrode type following initial conditioning. The SS arrays experienced a significant increase in CSC across the first 7 days of ~50%. Following this initial change ongoing electroactivity was stable. The CH coated SS electrodes were found to have a slow reduction in CSC across the entire study period, a behavior that has been previously reported for CP based coating materials (Green et al., 2013; Hassarati et al., 2014). The loss of electroactivity by the end of the study was an average of 30% of the initial CSC. This reduction is known to plateau as mobile ions, CP backbone and hydrogel chains within the system reach an equilibrium state (Yamato et al., 1995; Green et al., 2009, 2010). The change in CSC was not expected to be the result of the high frequency stimulation and this was reflected in the electroactivity of the passive controls. At the conclusion of the study passive CH coated controls were found to have an average CSC of 63.23 ± 8.17 mC/cm^2^, compared to the stimulated arrays with a final CSC of 60.04 ± 10.32 mC/cm^2^. Similarly passive SS electrodes were found to have a CSC of 0.23 ± 0.02 mC/cm^2^ at 42 days, compared to the stimulated electrodes with a final CSC of 0.29 ± 0.09 mC/cm^2^.

**Figure 10:**
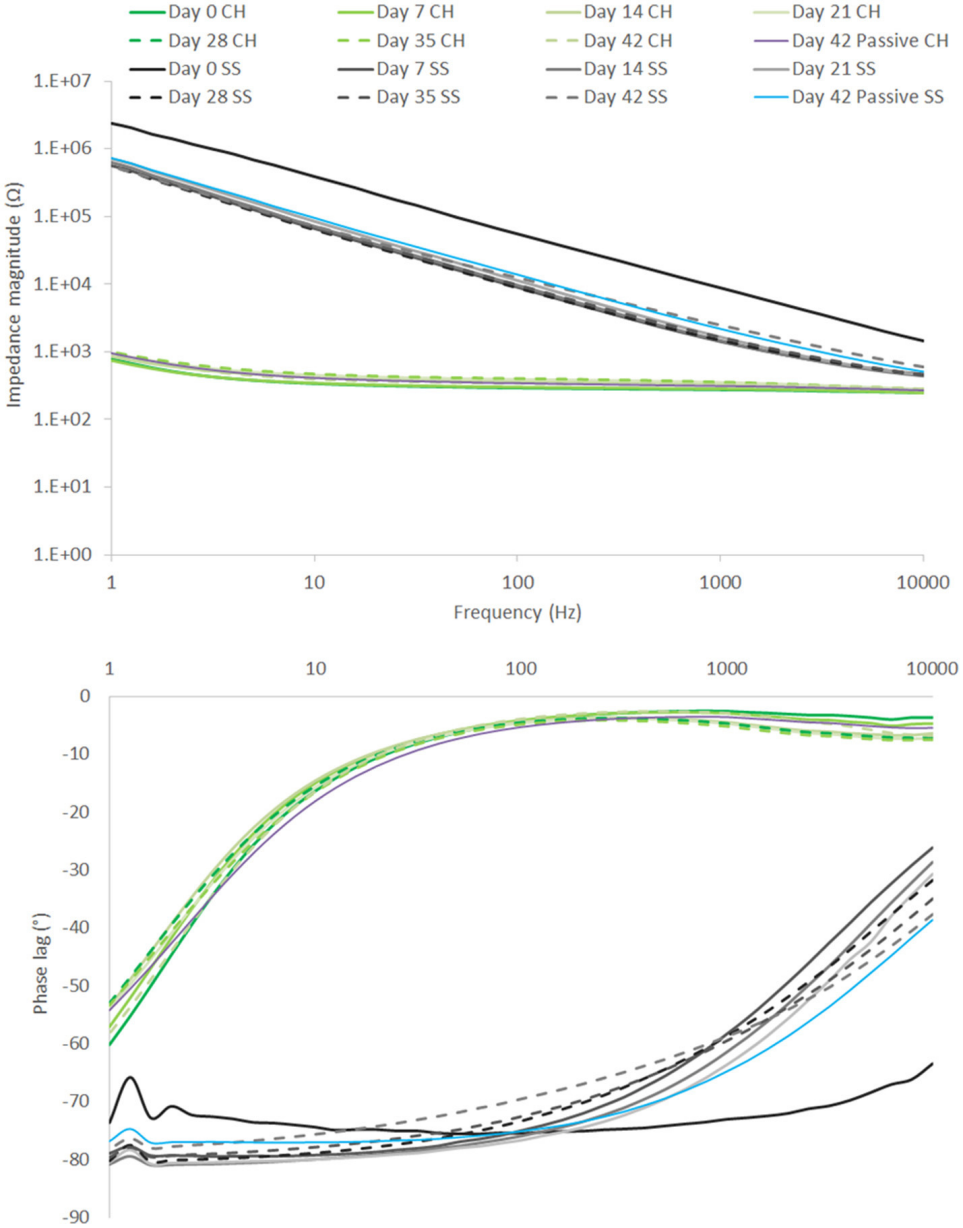
EIS over 42 day period of high frequency stimulation, showing performance of CH coated SS in comparison to uncoated SS. Passive controls are shown at termination of the study (*n* = 8).

**Figure 11:**
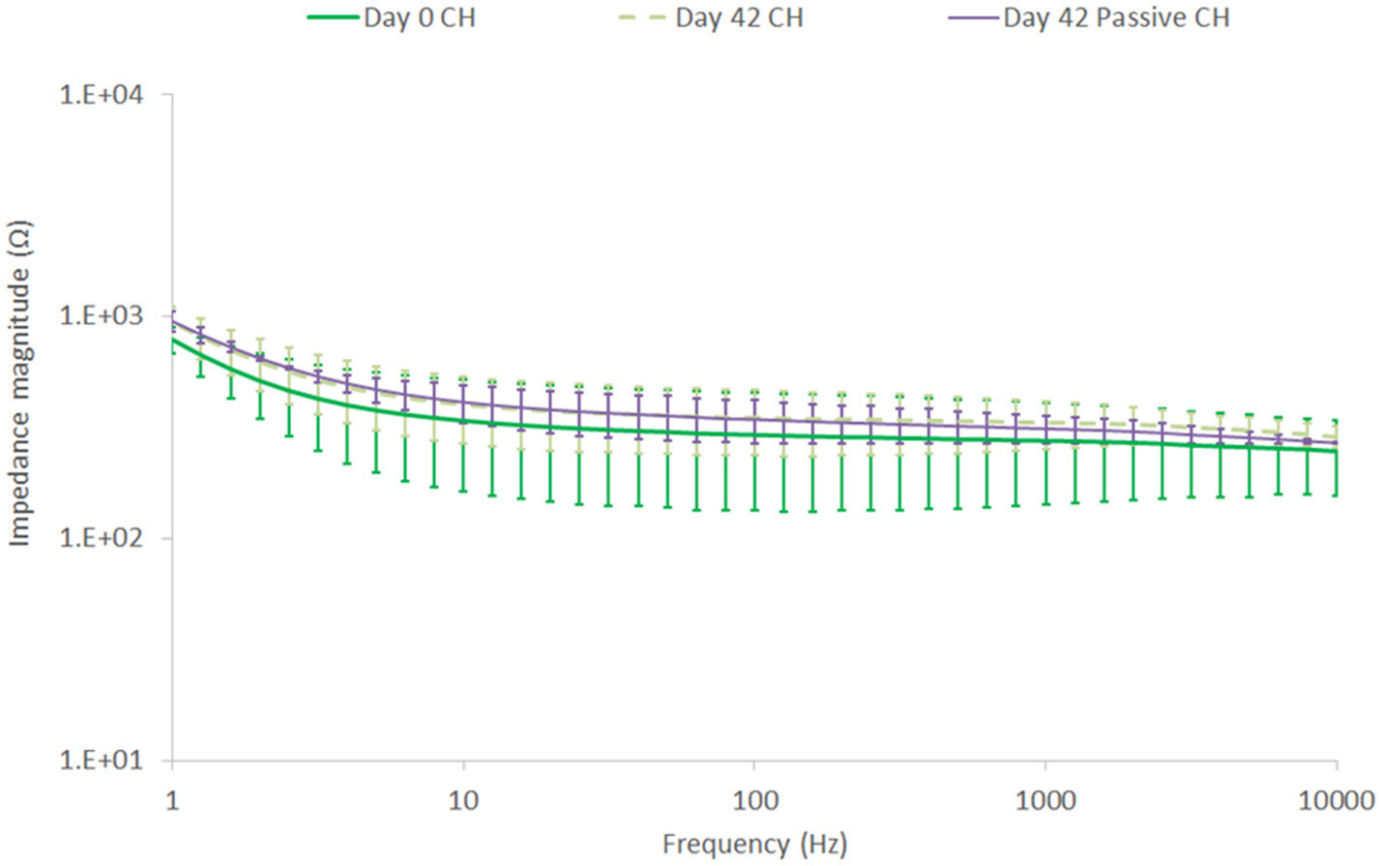
Impedance magnitude changes in the CH coating, comparing Day 0 to Day 42, unstimulated and stimulated at a high frequency of 40 kHz and 2 mA. Error bars are 1 SD, (*n* = 8).

EIS results depicted in Figure 10 reflect the same trend seen for the CV, with SS showing a substantial drop in impedance within the first 7 days, followed by continuous stable performance across the remainder of the study. The CH retains a stable EIS response, with a higher resolution image of the impedance magnitude seen in Figure 11. There is no significant difference in impedance across the study period and error bars are not shown in Figure 10, as they confound visualization of the data. One standard deviation was on average ± 100 Ω across all frequencies for the CH coated SS. Passive controls for both the SS and CH coated SS demonstrate that unstimulated controls performed similarly to the stimulated electrodes across this period. As such it is unlikely that the high frequency stimulation imparted any significant changes to either material. Finally, the electrode arrays were imaged at the conclusion of the study to establish cohesion of coating and any visible changes in electrode appearance. Neither electrode type was found to have notable changes (images not shown, no discernable difference to Figure 1).

## Discussion

CH coatings were shown to improve the performance of electrodes irrespective of the underlying metallic substrate. Both PtIr and SS electrodes were coated with a CH comprising the CP PEDOT and the hydrogel PVA-taurine. Despite these electrode arrays having significantly different initial properties, it was shown that the CH coating resulted in comparable electrochemical properties across both array types for a range of properties including CSC, impedance and charge injection limit. The electrode array format was shown to impact on both the electrochemical properties and device stability. The SS arrays were found to be stable under long-term high frequency stimulation and in particular maintained low impedance and high charge storage capacity when coated with the CH.

The majority of past studies into CH coated electrodes have been undertaken using Pt electrodes as the underlying substrate. It is feasible that the interaction between the CH coating and the underlying substrate may influence the electrochemical properties of the resultant electrode. CP coatings and hydrogel coatings have been applied to SS across a range of implants (Meng et al., 2009; Peixoto et al., 2009; Joung et al., 2012), however they are not commonly used in stimulating neuroprosthetics where CH coatings have been focused. In fact, much of the prior literature that encompasses hydrogel application to SS has been focused on imparting bioactivity to orthopedic implants and cardiovascular stents (Meng et al., 2009; Joung et al., 2012). In these applications chemical approaches have been used to covalently link coatings to SS or alternately the hydrogel used has been a degradable component employed for controlled drug elution. In this study, the electrochemically grown CP is used to anchor the coating to the implant electrodes. This forms an ionic association and mechanical interaction between the electrode and coating, but there is no covalent chemical bond. Nucleation of the CP at the electrode surface during polymerization will be critical to both electrochemical properties and long-term stability of the CH material (Arteaga et al., 2013; Patton et al., 2015, 2016). For the SS electrode array the cleaning protocol of repeat acid immersions and sonication was critical to reducing passivation and enabling CP deposition on these electrodes. Storage of the arrays within an oxygen accessible environment following cleaning, but prior to coating with the CP prelayer reduced the quality of the pre-layer and hence the overall CH coating. Alternately, the PtIr arrays were able to be coated with the same protocols used in prior literature for Pt electrodes. The stability of these arrays was rather impacted by the connections where exposed bonding of wires to the electrode pads led to failure of the device irrespective of the electrode coating.

The electrochemical properties of CH coatings were comparable to prior literature. The increase in CSC achieved by CH coating the SS arrays was 3 orders of magnitude and for the PtIr arrays was 2 orders of magnitude. Prior studies on CH coating of cochlear implants was found to produce electrodes with a CSC of 124 mC/cm^2^ (Hassarati et al., 2014), and CH coating of macroelectrodes (1 cm diameter discs) were found to have a CSC of 68.4 mC/cm^2^ (Green et al., 2012b) and 114 mC/cm^2^ (Goding et al., 2017). In these studies the CSC for the CH coated PtIr electrodes (212 mC/cm^2^) was almost double that of prior studies on device electrodes. The CH coated SS electrodes had an average CSC closer to that of the macroelectrodes (85 mC/cm^2^). As detailed in the Methods, both of these electrode types are relatively large, having dimensions on the mm scale, and as such could be expected to have properties closer to that of the macroelectrodes. Specifically, the PtIr electrodes have an area of 1.6 mm^2^ and the SS electrodes have an area of 0.11 mm^2^. It is know that charge transfer on microelectrodes is usually higher than that of macroelectrodes due to edge effects that enable higher charge density accumulation at the border regions (Cogan et al., 2016a). However, the PtIr electrodes have substantially higher CSC than both the smaller SS electrodes and macroelectrodes. It is feasible that the pre-curled cuff format influences the electrode behavior when testing by CV. Essentially the curled cuff will form a contained environment which restricts ion diffusion to the much larger volume of electrolyte in which the array is being tested. As a result, ions are likely to be sequestered within this volume and readily available for charge transfer during voltage cycling and associated redox reactions. This is supported by the EIS data which shows very low impedance for CH coated PtIr and also reduced impedance (per area) for uncoated electrodes at low frequency, where capacitive behavior through ionic double layer formation dominates electrical performance. It is important to know that this is a feature of the test system and would not be applicable to an in use cuff that is wrapped around a peripheral nerve.

The charge injection limits recorded in this study appear low when compared to prior literature reports for Pt and CP coated electrodes. This is likely due to the large size of these electrodes and the focus on shorter phase lengths (Rose and Robblee, 1990; Cogan et al., 2005). It is clearly seen in both this study and prior literature that there is a substantial phase dependence related to electrochemical charge injection limit (Green et al., 2012c, 2013, 2014). The majority of studies in the literature have focused only on a single phase length of 0.2 ms, being equivalent to pulse of 5 Hz. For comparison to literature, with a phase length of 0.2 ms the average injection limit of the CH coated SS was 223.3 μC/cm^2^, the CH coated PtIr was 122.8 μC/cm^2^, the bare SS was 6.2 μC/cm^2^, and the bare PtIr was 20.1 μC/cm^2^. At this same length of phase, literature has reported Pt microelectrodes without surface modification to have a charge injection limit within the range of 20–150 μC/cm^2^. Microelectrodes are known to have increased charge injection limit, as edge effects contribute to high charge density at the electrode surface. In a study by Green et al. (2012c) it was shown that Pt electrodes with a diameter of 1 mm produced a charge injection limit that was consistently below 30 μC/cm^2^ (across phase lengths varied from 0.1 to 0.8 ms) when tested in saline under identical conditions to the present study. However, as the focus of this study was high frequency neuromodulation the injection limit at phase lengths below 100 μs is critical. At 40 kHz the injection limit was increased by 5.5 times when SS electrodes were coated with CH and by four times when PtIr electrodes were coated with CH.

Long-term stimulation at 40 kHz and with 2 mA of peak to peak current was conducted using both SS and CH coated SS electrodes. The PtIr electrode arrays were unable to deliver this stimulation due to corrosive failure in the array connections. This was ultimately a factor of the electrode design and future studies will first seek to address areas of electrolyte leakage to the connections. At high frequency all materials act as resistors and the geometric surface area of the electrode is thought to dominate charge transfer behavior. The SS electrode designs have been used in impedance tomography for mapping of neural activity (Aristovich et al., 2016), which supports their use with high frequency pulses, however their stability under continual use for nerve block application was not known. These studies reveal that both SS and CH coated SS support stable delivery of pulses with no significant change in voltage drop across the 42 day period, during which more than 150 billion pulses were delivered. The CSC of the SS was found to increase across the initial period of stimulation and this was matched with a reduction in impedance magnitude and shift in phase lag. This behavior is not uncommon for metals that passivate, and the initial stimulation period is likely to have conditioned the surface such that charge transfer is more efficient (Williams and Williams, 1974; Miyazaki et al., 2005). Conversely the CH material was found to have a small increase in impedance and reduction in CSC. This is also not uncommon to conductive polymer based materials and known to plateau to yield stable electrochemical characteristics in the long-term (Yamato et al., 1995; Cui and Martin, 2003; Green et al., 2012a, 2013; Goding et al., 2017). The performance of the passive (unstimulated) controls, being not significantly different to that of the stimulated electrodes at 42 days, confirm that these changes in electrochemical performance did not occur as a result of stimulation. In fact, it is most likely that the rearrangement of polymer chains and the loss of mobile, unreacted components are largely responsible for this behavior. This degree of electrochemical stability has been reported for conventional PEDOT coatings before; Wilks et al. (2009) reported a high degree of electrochemical stability of PEDOT coatings on iridium-silicon microelectrodes under continuous biphasic pulsing. After 720,000 stimulation cycles there was no significant change in CSC or impedance, and only minor changes in the charge injection limit of the PEDOT coating. The findings presented in this paper demonstrate that the electrochemical stability of PEDOT is preserved in these CH coatings.

Ultimately, the CH coating reduced the potential transient required to drive the SS electrodes by 33% and maintained a significantly lower impedance and higher CSC across the study period. Since these studies were undertaken in saline without tissue contact, the full benefit of the CH coating has not been fully realized. It is expected that the reduction in stiffness imparted by the hydrogel will result in less scar tissue development over chronic implant periods, and the natural anti-fouling property of the hydrogel (Cheong et al., 2014; Hassarati et al., 2014) will minimize protein blocking at electrode sites. Future work will investigate the application of CH coated SS cuffs *in vivo* over both acute and chronic implant periods.

## Author contributions

NS and JG contributed equally as first authors in both experimental work and writing. AG, KA, and PB-P contributed to key aspects of the experimental work and provided input to the manuscript specific to their experimental contributions. DH, JM, and NL were co-investigators and contributed to study design and data analysis. DC was the major industry collaborator and contributed to study design and parameters. RG was the principal investigator contributing to study design, supervision, data analysis, and preparation of the manuscript.

### Conflict of interest statement

DC is an employee of Galvani Bioelectronics, the funder of these research activities. The other authors declare that the research was conducted in the absence of any commercial or financial relationships that could be construed as a potential conflict of interest.
